# Characterizing the initial effects of the single accreditation system merge on the ophthalmology residency match

**DOI:** 10.1080/10872981.2024.2307124

**Published:** 2024-01-23

**Authors:** Forrest Bohler, Allison Garden, Christian J. Santiago, Lily Bohler, Varna Taranikanti

**Affiliations:** a Department of Foundational Medical Studies, Oakland University William Beaumont School of Medicine, Rochester, MI, USA; b Edward Via College of Osteopathic Medicine - Carolinas Campus, Spartanburg, SC; c Montana State University, Bozeman, Montana

**Keywords:** Single accreditation merge, ophthalmology match, ophthalmology residency, Graduate Medical Education, ACGME, osteopathic medical students, allopathic medical students

## Abstract

**Introduction:**

In 2020, the American Osteopathic Association merged its residency programs into one system under the Accreditation Council for Graduate Medical Education (ACGME). The effects of this transition on the ophthalmology match is not fully understood. The purpose of this study is to assess the early impact of the transition to ACGME accreditation on MD, DO, and IMG representation in ophthalmology residency programs.

**Materials and Methods:**

Information about resident medical degree and resident medical school was gathered from ophthalmology residency program websites from a resident class before and after the Transition. Additionally, the medical degree of residency program directors (PD) was collected to analyze MD vs DO leadership in ophthalmology residency programs and to further stratify resident data to identify any trends in PD preference for different medical graduates.

**Results:**

Data was obtained for 915 ophthalmology residents in 110 residency programs that met the study’s inclusion criteria. Of these programs, 102 were allopathic with MD leadership, 1 was allopathic with DO leadership, 3 were osteopathic with MD leadership, and 4 were osteopathic with DO leadership. Overall, MD representation increased while DO and IMG representation decreased although not significantly. For both classes analyzed, DO and IMG representation was disproportionately low.

**Discussion:**

The transition to ACGME accreditation seems to have primarily harmed DO and IMG applicants in the ophthalmology match while benefitting MDs. Various factors such as loss of protected residency positions for DO applicants and the closure of osteopathic ophthalmology residency programs are likely reasons to blame for this decrease in osteopathic representation.

## Introduction

There are three primary routes to becoming a practicing physician in the United States (US). Two of these options involve receiving a domestic medical education from either an allopathic medical school (MD) or osteopathic medical school (DO). The third option involves attending an allopathic medical school outside of the US and then completing residency back in the US. These foreign graduates are collectively referred to as International Medical Graduates (IMG). The common thread among all three types of medical graduates is that they must complete residency within the US if they are to be allowed to practice medicine within the country.

Prior to 2020, medical graduates utilized two separate systems to apply to residency programs. MD graduates and IMGs participated in the National Residency Matching Program (NRMP) in order to apply to allopathic residency programs. DO graduates, on the other hand, could apply to allopathic residency programs through the NRMP but also had the option of applying to osteopathic residency programs under the American Osteopathic Association (AOA). While these residency programs were fewer in number compared to allopathic programs, they were considered protected residency positions for DO graduates as DO graduates were the only eligible candidates to apply for these positions. This dynamic changed in 2020 when the Accreditation Council for Graduate Medical Education (ACGME) finalized the transition that began in 2015 moving all residency programs into a single graduate medical education accreditation system (SGMEAS), completely eliminating the AOA match [1]. This meant that medical graduates of any degree type (MD, DO, or IMG) could apply to any US-based residency program. This action by the ACGME is now colloquially referred to as the ‘Transition.’

This was a highly controversial decision, especially among the osteopathic community, as the effects of the Transition remain largely unknown. Advocates of an SGMEAS claim that this streamlined application system would reduce the complexities of applying to both allopathic and osteopathic residency programs for DO graduates while also leading to greater standardization in residency curricula [2,3]. Additionally, proponents believe that an SGMEAS would increase DO representation and acceptance among allopathic residencies by decreasing stigma surrounding the DO medical degree. The benefits for MDs and IMGs were more apparent and immediate as the Merge gave access to osteopathic residencies that were once not available to these graduates [4]. Opponents of the Merge, however, were largely concerned with the negative ramifications for DOs. While MDs and IMGs have gained access to new residency positions, this has been at the expense of positions that were once exclusively reserved for DO graduates. NRMP data from 2020 revealed that MDs and IMGs secured 27.6% of these osteopathic residency positions [5]. Additionally, concerns regarding the ability of existing osteopathic residencies to meet the new accreditation requirements set forth by the ACGME have already been validated in part. Studies show that a significant number of residency programs in competitive surgical subspecialties, such as Otolaryngology and Ophthalmology, were ended due to an unwillingness or inability to achieve the new ACGME accreditation standards [6]. At the time of the announcement of the SGMEAS Transition in 2014, 15 osteopathic ophthalmology programs existed. By the time the Merge was fully implemented in 2020, this number had fallen to 7 programs, a 53.3% decline [6].

Each year, match data for ophthalmology is made publicly available through the San Francisco (SF) Match in coordination with the Association of University Professors of Ophthalmology’s summary report [7]. Although, this report contains data that reveals the total number of osteopathic and allopathic graduates successfully matching into ophthalmology residencies, it fails to provide further details regarding which type of ophthalmology residency program (osteopathic vs. allopathic) these graduates match into. This has made assessing the impact of the Transition on the ophthalmology match relatively difficult, and its effects remain largely unknown. Information regarding the specific match results of graduates into different program types could provide valuable insight into the underlying effects of the Transition among different program types.

The purpose of this study is two-fold. First, it is exploratory in nature and aims to assess the initial impact of the Transition on the ophthalmology match by investigating the change in representation of MDs, DOs, and IMGs for allopathic and osteopathic programs before and after the Transition. Second, it investigates any potential preferences for specific graduate medical degree types based on the medical degree held by residency program directors (PD).

## Methods

**Figure 1.**
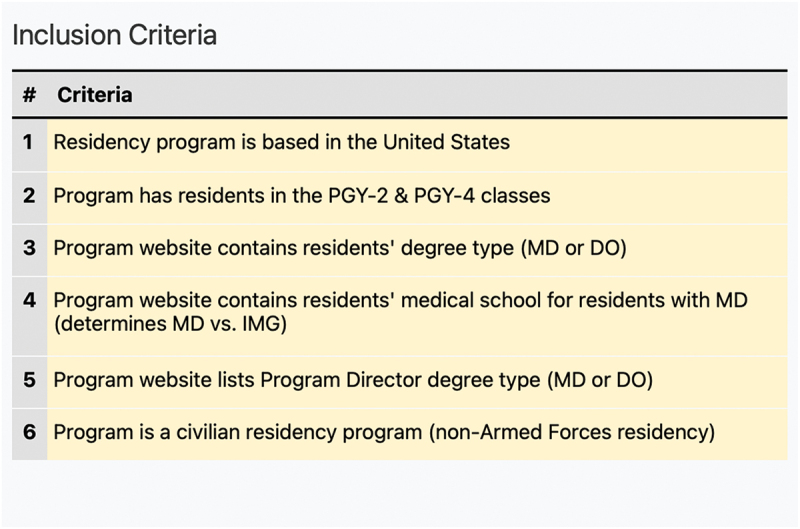
Inclusion criteria.

Using the Doximity Residency Navigator, 125 US-based ophthalmology residency programs were identified [8]. Seven osteopathic ophthalmology residency programs were identified using the findings from Ahmed et al. still training residents after the Transition [6]. Residency program websites were then utilized to collect the school at which residents received their medical education to determine if the resident was a foreign or domestic graduate. DO residents were assumed to be domestic graduates as there are no foreign osteopathic medical schools in existence. Resident degree type was then collected to determine the number of MD and DO graduates in each program. This information was collected for the PGY-4 class that matriculated into their residency program in 2019 (pre-Transition) and for the PGY-2 class that matriculated into their residency in 2021 (post-Transition). Data for the PGY-3 class was not collected or included in this study to reduce the impact that the COVID-19 pandemic may have had during the 2020 match year. Ophthalmology residency programs participate in an internship year for residents’ PGY-1 year. Many residency programs do not list PGY-1 resident information on their program website, so PGY-1 data was not included in this study. Programs that did not make pertinent resident information readily available on their program website for the PGY-2 and PGY-4 classes were excluded from this study. Armed Forces ophthalmology residency programs were also excluded from the study since these programs utilize a separate matching system. Figure 1 outlines the full inclusion criteria for programs in this study.

To further categorize residency programs, the degree held by each program’s PD was collected and residents were stratified by degree type and then placed in one of four residency categories: Allopathic with MD Leadership, Allopathic with DO Leadership, Osteopathic with MD Leadership, and Osteopathic with DO Leadership.

### Data analysis

The data was analyzed using descriptive statistics. The total number of residents for each degree type was calculated for the PGY-2 and PGY-4 classes. This number was then divided by the total number of residency programs that met the study’s inclusion criteria (110 programs) to find the mean number of MDs, DOs, and IMGs for the PGY-2 and PGY-4 years. This mean value for each degree type was compared between PGY-2s and PGY-4s within their respective program category using a Welch’s T-test with a statistical significance evaluated at 0.05 α level.

## Results

Of the 125 residency programs that were screened, data was collected for 110 programs which met the study’s inclusion criteria (Figure 1). Of these 110 programs, data was obtained for 455 PGY-4 residents and 460 PGY-2 residents. The results were analyzed in its entirety and also grouped into the following categories: Allopathic with MD Leadership, Allopathic with DO Leadership, Osteopathic with MD Leadership, and Osteopathic with DO Leadership.

**Figure 2.**
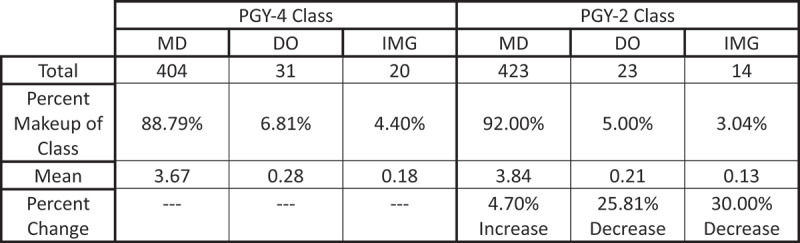
MD, DO, and IMG representation among all ophthalmology programs for the PGY-4 and PGY-2 classes.

In total, there were 404 MD-graduates in the PGY-4 class compared to 423 MD-graduates in the PGY-2 class. This difference of 19 MD residents reflects a 4.70% increase between the two classes after the Transition. There were 31 total DO-graduates in the PGY-4 class yet only 23 DO-graduates in the PGY-2 class, reflecting a 25.81% total decrease after the Transition. For IMGs, there were 20 in the PGY-4 class yet only 14 in the PGY-2 class, reflecting a 30.00% decrease after the Transition (Figure 2).

The percent distribution of degree type for the PGY-2 and PGY-4 classes is represented in Figure 2.

The mean number of MD-graduates per ophthalmology residency program increased between the PGY-4 to PGY-2 classes from 3.67 to 3.84 (*p* = 0.1749). The mean number of DO-graduates decreased between the PGY-4 to PGY-2 from 0.28 to 0.21 (*p* = 0.6861). The mean number of IMGs decreased between the PGY-4 to PGY-2 from 0.18 to 0.13 (*p* = 0.7338). These differences, however, were not significant (*p* < 0.05) (Figure 2).

### Breakdown of program types

**Figure 3.**
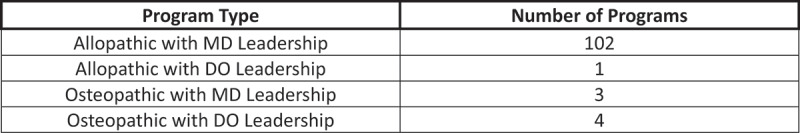
Overview of ophthalmology residencies by program type and leadership medical degree.

There was an uneven distribution of residency programs within the four categories. Of the 110 ophthalmology residency programs that met the study’s inclusion criteria, 102 were allopathic with MD leadership, and only 1 program was allopathic with DO leadership. Of the osteopathic residency programs, 3 had MD leadership and 4 had DO leadership (Figure 3).

### Allopathic programs

**Figure 4.**
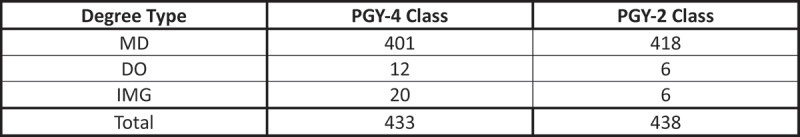
Overview of residents in allopathic with MD leadership residency programs for the PGY-4 and PGY-4 classes.

Allopathic programs with MD leadership experienced an increase in MD-graduates with a decrease in DO-graduates and IMGs. Both DO and IMG representation fell by a total of 6 residency positions, yet DO representation experienced a 50% decline from the PGY-4 class compared to a 30% decline for IMG representation (Figure 4). Additionally, allopathic programs with MD leadership was the only category that had any IMG representation for either PGY-4 or PGY-2 classes.

Although Cook County Health was the only Allopathic with DO Leadership, this program showed a greater level of diversity between MD and DO-graduates as its PGY-4 class contained 75% MD-graduates and 25% DO-graduates (3 MD residents and 1 DO resident) while the PGY-2 class was evenly split between MDs and DOs (2 MD residents and 2 DO residents).

### Osteopathic programs

Among the 7 osteopathic programs, 18 residency positions were available for each class. These 18 positions in the PGY-4 class were held exclusively by DO-graduates since this was before the Transition. In the PGY-2 class, DOs retained 15 of these positions while MDs secured 3 of these spots, representing a 16.67% increase in MD representation among all osteopathic residencies. No IMGs were accepted in the osteopathic programs.

**Figure 5.**
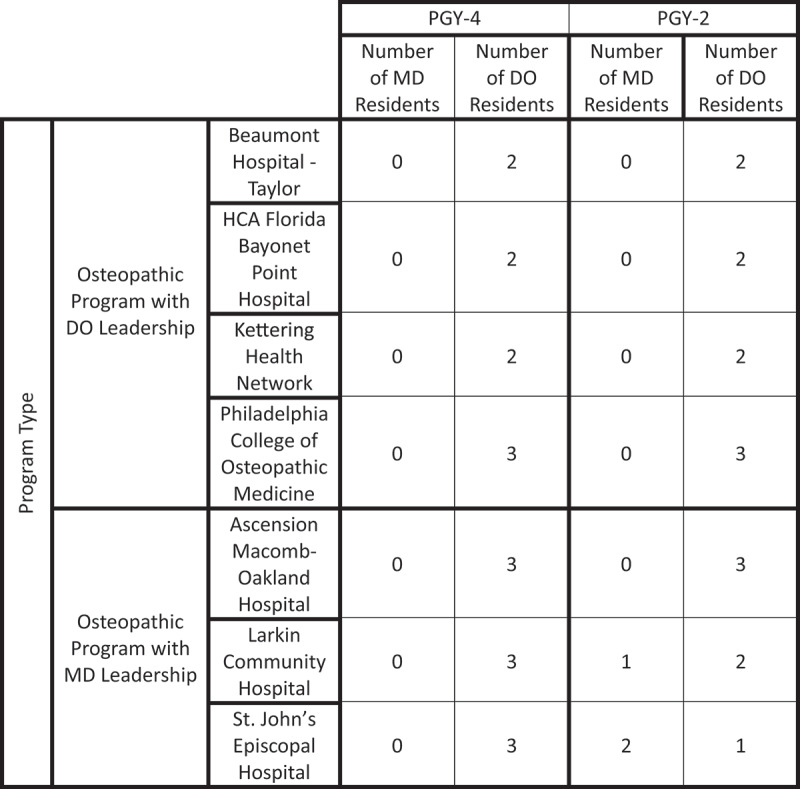
Overview of residents in osteopathic programs for the PGY-4 and PGY-2 classes.

Although there were 3 MD-led and 4 DO-led programs, there was an even distribution of residency positions available between the two groups (9 positions per group). There was an observable difference among the programs that accepted MD-graduates when stratified by the degree type held by PDs. The MD-led programs accounted for all MD-graduates in osteopathic residencies whereas no MD-graduates were accepted among the DO-led programs (Figure 5).

## Discussion

Overall, the initial effects after the Transition seem to have largely benefitted MD-graduates, as their total representation between the PGY-4 and PGY-2 classes has increased while at the expense of DOs and IMGs. DO representation fell between the two classes by 25.81%, whereas IMG representation experienced the greatest total percent decrease of 30.00%. DOs, however, experienced the greatest total loss in residency positions between the PGY-4 and PGY-2 classes. These observed differences between the PGY-4 and PGY-2 classes, however, were not significant.

There were notable selection patterns among programs that had leadership with degrees that differed from the program type. The 3 osteopathic programs with MD leadership and 1 allopathic program with DO leadership had the greatest diversity in representation of MD and DO-graduates, although they did not have any IMGs. IMG ophthalmology residents were only observed in allopathic programs with MD leadership for both PGY-2 and PGY-4 classes.

Although the total number of residency positions filled by IMGs fell in the PGY-2 class, DOs seem to have experienced the worst ramifications of the Transition. Despite the positive impact of the Transition on overall match results for DO-graduates as portrayed by the AOA [9], these benefits do not seem to apply to osteopathic graduates applying for ophthalmology residency, as the negative effects on DOs were three-fold in this specialty. First, DOs lost protected residency positions that were once exclusively available to them. In the PGY-2 class, MDs obtained 16.67% of these protected positions, although this percentage is lower than the national average across specialties in which 27.6% of these positions have been taken by MDs and IMGs [5]. Further, DO representation within allopathic programs decreased, contradicting proponents’ predictions that claimed DO representation would increase in allopathic programs after the Transition [10,11]. Finally, the closure of osteopathic ophthalmology programs since 2014 due to the new ACGME accreditation standards has further harmed DOs [6]. In total, DOs now represent over 25% of graduates in US medical school classes [12] yet makeup only 5.00% of total US ophthalmology residency positions in the PGY-2 class, indicating an underrepresentation of DOs in the specialty.

While some may attribute this underrepresentation to stigma held by PDs against the DO degree [13], there are other reasons that may explain this discrepancy. Ophthalmology is an extremely competitive specialty to match into. Traditionally, students’ United States Medical Licensing Examination (USMLE) Step 1 score has been one of the most important factors among PDs in the selection for their program [14]. Although the USMLE Step 1 exam has moved to a Pass/Fail model in recent years, the residents included in this study took this test prior to this shift meaning their scores were important factors in their selection for residency [15]. In addition, matched allopathic graduates on average tend to have scored significantly higher on this exam compared to their osteopathic counterparts with average scores of 233 and 227, respectively [16]. Given that their scores were included in their residency application, the potential discrepancy in Step 1 scores among MD and DO applicants could have affected their selection. In addition to excelling academically in pre-clinical and clerkship coursework, applicants must also have high achievement in extracurricular activities such as research, volunteering, and leadership, which are used to assess applicant competitiveness according to a survey of ophthalmology PDs [17]. There is a notable discrepancy in both research experiences and production (measure of abstracts/presentations/publications) between MDs and DOs. At the time of residency application, MDs on average have 4.0 research experiences, whereas DOs have only 2.2 experiences representing a 45% decrease [18]. Differences in research production are more profound as MD applicants on average have a production of 8.1 while DOs only have 3.4, representing a 57.5% decrease [18]. The additional time commitment to complete osteopathic manipulative medicine training may account in part for the lower engagement in research for osteopathic students. Another factor that may influence differences in competitiveness between degree types is related to the lack of home ophthalmology programs at most osteopathic medical schools. It is well established that access to a home residency program provides a wealth of specialty-specific networking opportunities for students. These networking opportunities can often times lead to ophthalmologist-written letters of recommendation (LORs) as well as increased perceived commitment to the specialty, both important factors among PDs within ophthalmology and across all specialties in general when selecting applicants for interviews [14,17]. In theory, an applicant that had access to a home ophthalmology program will have LORs that are greater in number and stronger in nature than applicants without home programs. Therefore, since osteopathic medical schools have disproportionately fewer home ophthalmology programs, they are at a distinct disadvantage when trying to increase their competitiveness.

Osteopathic institutions also emphasize primary care within their curricula. This emphasis may influence its students away from subspecialties while also self-selecting their student population who may have entered medical school with a greater interest in primary care. This could contribute to the disproportionately low representation in the specialty. Further qualitative studies that investigate osteopathic medical students’ desires and interest to enter surgical subspecialties could address this question. Additionally, year-to-year fluctuations in interest among DO applicants could also be responsible for the decline in DO representation in the post-Transition class but is unlikely given the number of DO applicants remained relatively the same during both residency classes (44 DO applicants within the PGY-2 class vs. 45 DO applicants within the PGY-4 class) [7].

In addition to a disproportionately low number of osteopathic residents in ophthalmology programs relative to the number of osteopathic medical graduates, osteopathic leadership was also disproportionately low. Of the 110 residency programs analyzed, only 5 had PD with a DO medical degree. These 5 PDs represent 4.54% of all ophthalmology PDs, which is strikingly similar to the 5% of PGY-2 ophthalmology residents with a DO degree. Perhaps, efforts to increase DO leadership in residency programs as well as having consistent evaluation standards for MD, DO, and IMG applicants would lead to proportionate representation in ophthalmology residency programs relative to the percentage of allopathic and osteopathic medical graduates.

Although not significant, there does seem to be a selection bias for concordant applicant degree type in relation to the degree held by PDs. This bias may be related to a stigma held against the DO degree, but it is equally likely that the degree held by PDs influences what attributes in an applicant makes them desirable to that PD. For example, PDs with MD degrees may value research more heavily than PDs with DO degrees. This could account for the potential bias suggested by the results of this study.

In order to address the lack of DO representation in ophthalmology programs, osteopathic medical institutions should place greater emphasis on research participation and early exposure to the field of ophthalmology, which will increase students’ competitiveness on par with MD applicants. If these institutions are unable or unwilling to make these adjustments, osteopathic students must take action on the individual level. At minimum, these students must be keenly aware of the uphill battle they face if they hope to match into an ophthalmology residency position. These students should focus on engagement in research and networking opportunities within the specialty early in their education, which may improve the chances of matching.

### Limitations

Our study has several limitations. First, the findings of our study reveal changes in the ophthalmology match after the Transition but cannot provide definitive reasons for these changes. We have attempted to provide some possible causes that explain these observations but further studies are needed to investigate the potential mechanisms behind these changes. One other potential reason for these observed changes could be due to random chance. This is unlikely, however, as other studies have revealed similar declines in DO representation among competitive surgical subspecialities such as orthopedic surgery, neurological surgery, plastic surgery, and thoracic surgery indicative of a pattern of decline rather than random chance [5,19]. Subsequent follow-up studies that investigate future ophthalmology residency classes are needed to make this determination, however.

Second, the Transition was fully implemented as the COVID-19 pandemic hit the US. This resulted in a shift to a virtual interview platform for most programs during the PGY-2 class. The PGY-4 class, however, which matriculated before the pandemic hit, mostly utilized in-person interviews. The effects of this move to a virtual format are still largely unknown and most effects remain speculative in nature but could be multifaceted. While the move from in-person to virtual interviews expanded accessibility for applicants to attend interviews, this meant it could be more difficult for applicants to convey their personal attributes not readily apparent within their applications. Since programs were unable to meet residents in-person during the interview season, they may have been more inclined to accept applicants that had conducted rotations through their programs. Most US medical schools with ophthalmology programs are allopathic [12]. This a greater advantage to MD-candidates who on average have more access to home ophthalmology program and can rotate through these programs. This could have potentially contributed to the increase in MD-graduates in the PGY-2 class.

## Conclusion

The results from this study reveal a non-statistically significant decrease in DO ophthalmology residents that may be associated with the Transition as the early impacts of the SGMEAS Transition for the ophthalmology match seem to have negatively affected DOs and IMGs while benefitting MD-graduates. The reasons for this decline are likely multifactorial which include loss of protected residency position in osteopathic programs, lower research production for DO applicants leading to a potential decline in overall acceptance, lack of home osteopathic ophthalmology programs leading to less competitive applicant profiles, and the shift to a virtual interview format during the pandemic, which may have given advantages to MD applicants.

Hence, we hope that the findings of this study will help all stakeholders in addressing the problems mentioned previously. Future studies should be conducted to evaluate the effects of the Transition in order to determine if the findings in this study were an anomaly or the new norm. Alternatively, the SF Match should consider releasing match data prior to the Transition to the public as the NRMP does so that this information would be easily accessible, circumventing the need for studies such as this.
